# All-optical modulation of quantum states by nonlinear metasurface

**DOI:** 10.1038/s41377-022-00744-5

**Published:** 2022-03-11

**Authors:** Di Zhang, Yang Chen, Shengchao Gong, Wei Wu, Wei Cai, Mengxin Ren, Xifeng Ren, Shuang Zhang, Guangcan Guo, Jingjun Xu

**Affiliations:** 1 The Key Laboratory of Weak-Light Nonlinear Photonics, Ministry of Education, School of Physics and TEDA Applied Physics Institute, Nankai University, Tianjin, China; 2 Key Laboratory of Quantum Information, CAS, University of Science and Technology of China, Hefei, China; 3 Synergetic Innovation Center of Quantum Information & Quantum Physics, University of Science and Technology of China, Hefei, China; 4 Collaborative Innovation Center of Extreme Optics, Shanxi University, Taiyuan, China; 5 Department of Physics, The University of Hong Kong, Hong Kong, China

**Keywords:** Metamaterials, Nanophotonics and plasmonics, Nonlinear optics

## Abstract

Metasurfaces have proven themselves an exotic ability to harness light at nano-scale, being important not only for classical but also for quantum optics. Dynamic manipulation of the quantum states is at the heart of quantum information processing; however, such function has been rarely realized with metasurfaces so far. Here, we report an all-optical dynamic modulation of the photonic quantum states using the nonlinear metasurface. The metasurface consists of a metallic nanostructure combined with a photoisomerizable azo layer. By tuning the plasmonic resonance through optically switching the azo molecules between their binary isomeric states, we have realized dynamic control of transmission efficiencies of orthogonally polarized photons and also the phase delay between them, thereby an entangled state was efficiently controlled. As an illustration, a quantum state distillation has been demonstrated to recover a Bell state from a non-maximally entangled one to that with fidelities higher than 98%. Our work would enrich the functions of the metasurface in the quantum world, from static to dynamic modulation, making the quantum metasurface going practical.

## Introduction

Nonlinear metasurfaces have recently captured an intense interest. Compare to the passive ones showing fixed optical properties, the response of the nonlinear metasurfaces can be tuned by external optical stimuli in an active manner. Such a dynamic framework provides new degrees of freedom to manipulate light behaviors in the time domain, and facilitates a transition from the traditional static metadevices to their dynamic counterparts^1,2^. In this context, various fascinating all-optical modulating functions have been demonstrated, including light phase modulation^3^, nonlinear polarization conversion^4^, spectral tuning^5^, spatial light modulations^6^, and so on^7^. However, all these dynamic functions were mainly motivated by the applications for classical light. On the other hand, the interest in applying metasurfaces to quantum optics has also been growing rapidly since the first experimental verification that photon entanglement survives through plasmonic nano-hole arrays^8–10^. Various remarkable success has been demonstrated^11^, including generation of entangled multi-photons^12–14^, reconstructing entangled states^15^, quantum edge detection using dielectric metasurfaces^16^, coherent perfect absorptions of single photons^17^, and so on. However, until now the above quantum metasurfaces are mostly static in nature. The dynamic functions of metasurfaces have rarely been used for manipulating photonic quantum states, which are beneficial to construct reconfigurable quantum information systems with a compact profile in the future.

Quantum information technique holds potential supremacy over classical technologies in computational efficiency and communication security. One of the most basic concepts and important resources for quantum optical applications is entangled photon pairs of the form^18^

1 $$\left| {\Psi} \right\rangle = \frac{1}{{\sqrt {\left| \alpha \right|^2 + \left| \beta \right|^2} }}\left( {\alpha \left| {V_S} \right\rangle \left| {V_I} \right\rangle + \beta \left| {H_S} \right\rangle \left| {H_I} \right\rangle } \right)$$

where $\left| V \right\rangle$ and $\left| H \right\rangle$ represent the vertical and horizontal polarization state of a photon, subscripts *S* and *I* represent the signal path and idle path, and *α* and *β* are complex amplitude of $\left| {V_S} \right\rangle \left| {V_I} \right\rangle$ and $\left| {H_S} \right\rangle \left| {H_I} \right\rangle$ components, respectively. The manipulations of such entangled states are fundamental in quantum algorithms for quantum computation and quantum communications^19–21^. In general, maximally entangled quantum states (*α*/*β* = 1) are required. However, owing to decoherence and dissipation in the quantum system, practical states normally degraded to be less entangled ($\alpha /\beta \ne 1$). Thus, it is essential to recover the maximally entangled states from these imperfect ones. Quantum entanglement distillation is one important method to settle this problem, which transforms *N* copies of an arbitrary entangled state into some number of approximately pure Bell pairs using only local operations and classical communication^22–25^. It has been reported that anisotropic structured metasurfaces can be used in quantum state distillation by employing their polarization-dependent transmission efficiencies. However, those metasurfaces were still static, and can only compensate for certain non-maximally entangled quantum states without any tunable ranges, which is apparently inconvenient for practical use^26^.

**Fig. 1:**
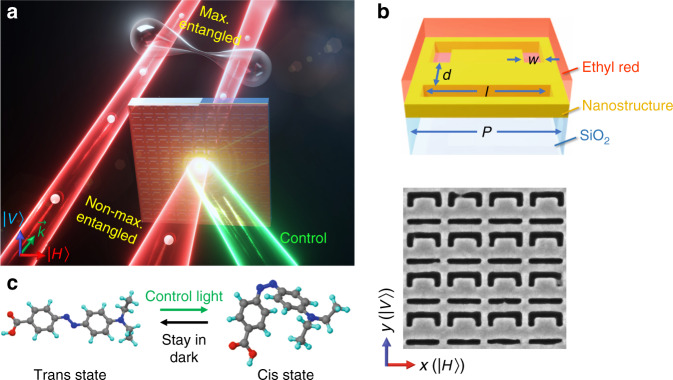
Dynamic control of entangled quantum states using a nonlinear metasurface. **a** The polarization-entangled two-qubit state is described by $\left| {{{\mathrm{{\Psi}}}}} \right\rangle = \frac{1}{{\sqrt {\left| \alpha \right|^2 + \left| \beta \right|^2} }}\left( {\alpha \left| {V_S} \right\rangle \left| {V_I} \right\rangle + \beta \left| {H_S} \right\rangle \left| {H_I} \right\rangle } \right)$. The metasurface is acting on only one of the photons. The nonlinear modulation of the quantum state $\left| {{{\mathrm{{\Psi}}}}} \right\rangle$ is accomplished by external control light with power $P_{{{\mathrm{C}}}}$ (green beam). $\left| {{{\mathrm{{\Psi}}}}} \right\rangle$ is not maximally entangled when $\alpha /\beta \ne 1$. The state distillation corresponds to the case of recovering $\alpha /\beta$ to 1. **b** Schematic of a single unit cell. Gold film with 45 nm thickness was sputtered onto a fused silica substrate. An array of anisotropic structured ASRs was patterned by FIB milling. A nonlinear switching layer of ethyl red polymer film was spin-coated on top. The designed geometric parameters are the period $P = 300\,{{{\mathrm{nm}}}}$, the slit length $l = 250\,{{{\mathrm{nm}}}}$, the slit width $w = 45\,{{{\mathrm{nm}}}}$, and the gap between “C” arm and “I” slit $d = 95\,{{{\mathrm{nm}}}}$. Representative SEM image of the sample is given in bottom panel. Vertical (*V*) and horizontal (*H*) directions are defined as along *y*- and *x*-axes, respectively. **c** The ethyl red molecule isomerizes from trans state to cis state by green-light irradiation, and then recovers to the original state through thermal relaxation in dark. Such reversible structural transformation would change the optical response of the metasurface, hence the value of $\alpha /\beta$

In this paper, we go beyond the above static metasurface scenario, and present the nonlinear metasurface for all-optical manipulation of the polarization-entangled photon states, and more specifically, the task of entanglement distillation, as shown by the schematic in Fig. 1(a). The metasurface consists of an anisotropic structured nanostructure layer covered by a photoisomerizable ethyl red film. By optically switching the transmission contrast and phase retardation between the orthogonally polarized photons through the metasurface, the states of polarization-entangled photon pairs were controlled efficiently. A non-maximally entangled state was recovered to that with fidelities higher than 98%. Our results open up a new horizon towards reconfigurable quantum systems and have the potential for realizing integrated on-chip quantum applications in the future.

## Results

Our metasurface consists of an array of asymmetric split ring (ASR) slits, which were manufactured by focused ion beam (FIB) milling through a 45 nm thick gold film rested on a fused silica substrate. The total array occupies a footprint of 21 × 21 μm^2^. Schematic of one unit cell and scanning electron microscope (SEM) image are illustrated in Fig. 1(b). The length and the width of the slits are $l = 250\,{{{\mathrm{nm}}}}$ and $w = 45\,{{{\mathrm{nm}}}}$, respectively, and the gap *d* between “C” and “I” slits is 95 nm. The lattice constant of the array $P = 300\,{{{\mathrm{nm}}}}$ is much smaller than the wavelength of the photon quanta used in our experiments (808 nm), so that photons can transmit through the metasurface without diffraction. Furthermore, the polymer film (mixture of ethyl red and polymethylmethacrylate, see method) was spin-coated on top of the sample, which was used as the nonlinear optical switch layer. Upon external control laser excitation (532 nm in our experiment), the molecular chains of the ethyl red would fold, leading to a structural transformation from trans state to cis state. By removing the optical stimulus, the ethyl red molecules return to the initial trans state through the relaxation process. Such molecular folding causes a decrease in the refractive index of the polymer film, thereby changing the optical response of the metasurface in a reversible manner.

**Fig. 2:**
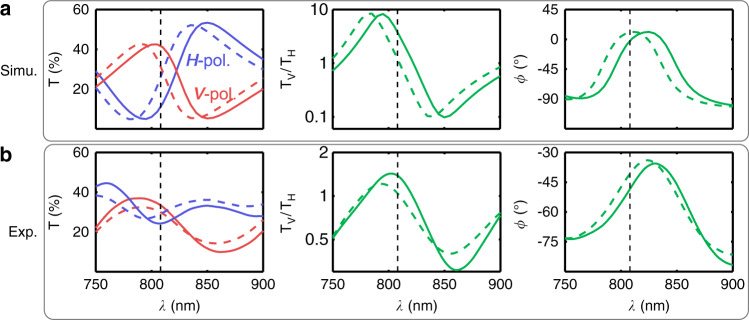
Nonlinear tuning of the optical response of metasurface. **a** Numerically simulated transmission *T* for *V*- and *H*-polarizations are given by red and blue lines, respectively. The anisotropic response is further characterized by the transmission ratio ($T_V/T_H$, the vertical coordinate is in log scale) and the relative transmittance phase difference *ϕ* between the orthogonal polarizations. Solid lines present results for metasurface without control light, and dashed lines are those under the control light. **b** Experimental results of *T*, $T_V/T_H$, and *ϕ*. The wavelength of photonic quanta is 808 nm, which is represented by vertical black dashed lines

The ASR is intrinsically anisotropic in structure, thus the metasurface would respond differently to the photons with orthogonal polarizations, leading to different transmission spectra *T*. We first performed numerical studies of the optical properties of the sample using a finite element method (COMSOL Multiphysics). The refractive indices of the quartz substrate, gold, and ethyl red used in simulations were adopted from ellipsometric measurements (Fig. S3 in the Supplementary information). The transmission spectra for vertically (*V*) and horizontally (*H*) polarized incidence without the control light are given by solid lines in Fig. 2(a). The metasurface shows a transmission valley around 800 nm and a peak near 850 nm for *H*-polarized incidence, while the *V*-polarized spectrum presents swapped wavelength positions for valley and peak. The transmission ratio ($T_V/T_H$) and relative phase delay *ϕ* between *V*- and *H*-polarizations are given in the right two panels of Fig. 2(a). The role of the nonlinear excitation by the control light was considered in the simulation by reducing the refractive index of the ethyl red film by 0.05^4^. The results are indicated by dashed lines in Fig. 2(a), and it is obvious that the spectra *T*, along with the transmission ratio $T_V/T_H$ and the phase delay *ϕ*, show distinct blue-shifts by control light excitation, leading to an efficient modulation to the quantum state $\left| {{{\mathrm{{\Psi}}}}} \right\rangle$ described in Eq. (1).

To experimentally characterize the properties of the metasurface, we built a spectrometer system to measure the transmittance *T* and the relative phase *ϕ* between *V*- and *H*-polarizations (Section I of the Supplementary information). A halogen lamp was used as the light source, whose output was collected by an objective (20×, 0.4 N.A.) to illuminate the sample. Another objective (20×, 0.45 N.A.) was used to collect the transmitted light, which was finally analyzed by a spectrometer (HR4000, Ocean Optics). A polarizer was inserted before the sample to polarize the incident light along a specific direction. To measure the transmittance of the *V*-polarization, the first polarizer was set vertically, which was again aligned horizontally for measuring the *H*-polarization. By further setting the incident polarization along +45° and analyzing the spectra for the ±45°, oppositely-handed-circular polarization components in the transmission, the relative phase *ϕ* between *V*- and *H*-polarizations was obtained. To stimulate the nonlinear response of the sample, a 532 nm continuous-wave (CW) laser (MGL-III-532, CNI Corp.) was used as the control light. The green light had a power *P*_*C*_ of 20 mW, and was focused down to a diameter of about 13 μm, leading to the light intensity of about 15.1 kW cm^−2^. The green light was combined with the halogen light using a polarization-independent beam splitter (BS). The measured spectra are given in Fig. 2(b), which match reasonably well with the simulated results in Fig. 2(a). However, the experimental results are flatter than simulations, which can be mainly contributed to the imperfect fabrications of the sample (Section II of the Supplementary information). Under the stimulus of the green light, the spectra of *T* and *ϕ* all experience blue-shifts as expected. Such spectral shifts lead to modification to both the value of $T_V/T_H$ and *ϕ* at a certain wavelength, such as 808 nm as indicated by vertical black dashed lines, resulting in a nonlinearly tunable *α*/*β*. Consequently, the quantum states in Eq. (1) could be controlled.

**Fig. 3:**
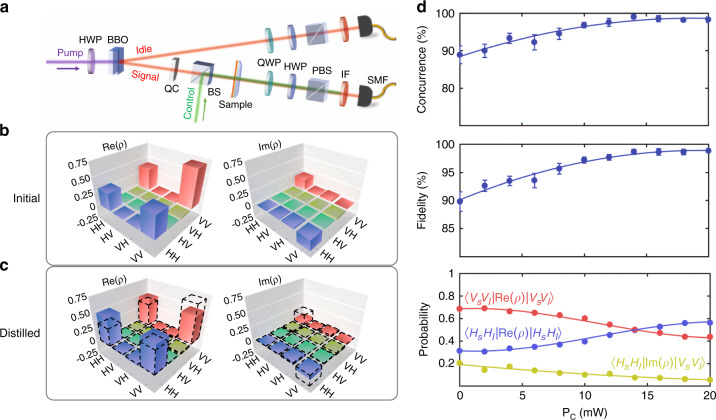
Dynamic control of quantum states. **a** Experimental setup for dynamic quantum state control. BBO: *β*-barium borate crystals; QC: quartz crystal; QWP: quarter-waveplate; HWP: half-waveplate; BS: beam splitter; PBS: polarized beam splitter; IF: interference filter; SMF: single-mode fiber. **b** The density matrix *ρ* of the initial state. The system has been biased to generate a non-maximally entangled state. The real part of *ρ* is given in the left column, and the imaginary part is shown on right. **c** The density matrix *ρ* of the distilled state after applying 14 mW control light. The initial state is redrawn here by dashed lines for a clear comparison. **d** The evolution of the quantum state as a function of the control light power ($P_{{{\mathrm{C}}}}$). Concurrence (top), fidelity (middle), and three characteristic elements of the density matrix (bottom) show the monotonic relationship with the power of the control light. Dots are experimental results, and lines are eye-guides

To illustrate the nonlinear control over the photonic quantum states by the metasurface, we replaced the above halogen lamp with a quantum light source. As depicted in Fig. 3(a), a 404 nm CW laser (MLL-III-404, CNI Tech., 200 mW) was used to pump a pair of type-I phase-matched *β*-barium borate crystals (BBO, CASTECH Inc.), which were stacked together with their optical axes along *H*- and *V*-directions respectively. *VV*-polarized (*HH*-polarized) photon pairs at the wavelength of 808 nm were generated on the first (second) BBO crystal via a process of spontaneous parametric down-conversion (SPDC). Subsequently, the entangled state of $\left| {{{\mathrm{{\Psi}}}}} \right\rangle = \frac{1}{{\sqrt 2 }}\left( {e^{i\varphi }\left| {V_S} \right\rangle \left| {V_I} \right\rangle + \left| {H_S} \right\rangle \left| {H_I} \right\rangle } \right)$ obtains. *φ* is determined by the phase matching condition and the BBO crystal thickness, and can be compensated to 0 by a birefringent quartz crystal (QC). Thus, the generated entangled state by type-I BBO crystals shows $\left| {{{{\mathrm{{\Psi}}}}}_0} \right\rangle = \frac{1}{{\sqrt 2 }}\left( {\left| {V_S} \right\rangle \left| {V_I} \right\rangle + \left| {H_S} \right\rangle \left| {H_I} \right\rangle } \right)$^27^. The twin photons traveled along two paths separately [labeled as signal and idle paths in Fig. 3(a)]. The metasurface was inserted between the two objectives in the signal path. Before coupling into single-mode fibers (SMFs) for spatial mode selection, the photons passed through a series of quarter-waveplate (QWP), half-waveplate (HWP), polarizing beam splitter (PBS), and interference bandpass filters (IFs) placed in respective paths. The photon pairs were then detected by avalanche photodiodes (APDs, EXCELITAS Tech.). The output signals from the two detectors are combined electronically in a time-correlated single-photon counting system (PicoHarp 300, PicoQuant GmbH), whose integration time was set as 10 s, and the coincidence window was 4 ns. The post-adjusted electronic time delay was applied to compensate for the non-equivalent optical path length between the two paths. The losses of the metasurface will cause the drop of photon counts in the signal path, thus decreasing the coincident counts of photon pairs proportionally. Because of the nonlocal property of the quantum entangled state, any local operation (i.e., operation on one photon) is equivalent to operating the whole entangled state^28^. We characterized the generated quantum states following standard quantum state tomography (QST)^29^, in which the measurement basis states $\left| H \right\rangle$, $\left| V \right\rangle$, $\left| D \right\rangle = \left( {\left| H \right\rangle + \left| V \right\rangle } \right)/\sqrt 2$, and $\left| R \right. = \left( {\left| H \right. + i\left| V \right.} \right)/\sqrt 2$ were chosen by selective combinations of the aforementioned QWP, HWP, and PBS. Hence the density matrix *ρ*, concurrence, and fidelity of the quantum states can be derived (Section III of the Supplementary information).

To demonstrate the entanglement distillation by the nonlinear metasurface, the initial photon state (including the effect of the sample) was deliberately generated as a non-maximally entangled one $\left| {{{\mathrm{{\Psi}}}}} \right\rangle = \frac{1}{{\sqrt {\left| \alpha \right|^2 + \left| \beta \right|^2} }}\left( {\alpha \left| {V_S} \right\rangle \left| {V_I} \right\rangle + \beta \left| {H_S} \right\rangle \left| {H_I} \right\rangle } \right)$ ($\alpha /\beta \ne 1$) by adjusting the 404 nm HWP and QC compensators. The measured density matrix *ρ* of this initial state is shown in Fig. 3(b), in which the left panel is the real part [Re(*ρ*)], and the right one is the imaginary part [Im(*ρ*)]. Four corner components show unbalanced values. Furthermore, the concurrence and fidelity (with respect to the Bell state) were calculated as 88.9 ± 2.4% and 89.8 ± 1.7%, respectively [as shown by the data points of *P*_C_ = 0 in Fig. 3(d)]. Then the all-optical nonlinear entanglement distillation process was performed by irradiating the sample with the 532 nm laser. As a result of the nonlinear change of the transmission ratio $T_V/T_H$ and relative phase *ϕ* between *H*- and *V*-photons, the quantum state is changed accordingly. The density matrix *ρ* of the distilled quantum state is shown in Fig. 3(c). Compared with the initial state (redrawn here by dashed lines for a clear comparison), the four corner elements in the Re(*ρ*) matrix tend to be balanced, and two anti-diagonal elements of Im(*ρ*) approach zero, confirming the applicability of nonlinear metasurface arrays for the state distillation and the recovery to the Bell state $\frac{1}{{\sqrt 2 }}\left( {\left| {V_S} \right\rangle \left| {V_I} \right\rangle + \left| {H_S} \right\rangle \left| {H_I} \right\rangle } \right)$ (for $\alpha /\beta = 1$). As shown in Fig. 3(d), the concurrence, fidelity, and certain characteristic corner elements of the *ρ* matrix all vary monotonically when the power of the control light *P*_C_ was gradually increased to 20 mW. The concurrence and fidelity rise to 99.1 ± 0.4% and 98.7 ± 0.5% for $P_{{{\mathrm{C}}}} = 14\,{{{\mathrm{mW}}}}$, and become saturated for larger *P*_C_. The *ρ* matrix element $\left\langle {H_S} \right|\left\langle {H_I} \right|{{{\mathrm{Re}}}}\left( \rho \right)\left| {H_S} \right\rangle \left| {H_I} \right\rangle$ becomes equal to $\left\langle {V_S} \right|\left\langle {V_I} \right|{{{\mathrm{Re}}}}\left( \rho \right)\left| {V_S} \right\rangle \left| {V_I} \right\rangle$ at $P_{{{\mathrm{C}}}} = 14\,{{{\mathrm{mW}}}}$; however, they deviate from each other for larger *P*_C_ due to over-modulation. On the other hand, the $\left\langle {H_S} \right|\left\langle {H_I} \right|{{{\mathrm{Im}}}}\left( \rho \right)\left| {V_S} \right\rangle \left| {V_I} \right\rangle$ approaches zero monotonically as *P*_C_ increases.

## Discussion

In conclusion, we have realized the dynamic all-optical control of the photonic quantum states using the nonlinear metasurface. Specifically, the function of entanglement distillation was illustrated, which is essential to settle the problem of quantum state degradation by decoherence and dissipation in the practical quantum system. A non-maximally entangled state has been successfully recovered to the ones with concurrence and fidelity higher than 99% and 98%, respectively. Compared with the mechanical method^30^, and other nonlinearities such as Kerr effect^31,32^ or thermo-optical effect^33–35^, the photoisomerization process demonstrated here has the advantages of low pumping power (only needs milliwatts CW pump power) and fast response time (on the order of milliseconds, Section V of the Supplementary information)^4^. Thus, utilizing a feedback loop to control the pump light power of our metasurface, a novel adaptive quantum optical source with the optimum degree of entanglement may be built. Furthermore, apart from entanglement distillation, we believe our nonlinear metasurface framework can be extended to accomplish more complex quantum state engineering tasks, such as logic gates in quantum information processing and quantum computations. The nano-scale sizes of metasurfaces also make them appealing to construct integrated quantum systems.

## Materials and methods

### Fabrication of the metasurface

A 45 nm Au layer was sputtered on a fused silica substrate. The nanostructures were patterned by FIB milling under 30 kV Gallium beam and the beam current was 1.8 pA. The overall area of the sample is about 21 × 21 μm^2^. The ethyl red solution was made by dissolving ethyl red powder in ethanol with the concentration of about 18 wt-%. Then the solution was mixed with polymethylmethacrylate (PMMA) at the volume ratio of 1:1. The mixture was spin-coated on top of the metasurface at the speed of 1500 rpm, which formed a polymer film with the thickness of about 300 nm.

## Supplementary information

Supplementary information for All-optical modulation of quantum states by nonlinear metasurface
