# Supplementary material for: All-optical modulation of quantum states by nonlinear metasurface
Source: Light Sci Appl. 2022 Mar 11;11:58. doi: 10.1038/s41377-022-00744-5 (PMC8917209; doi:10.1038/s41377-022-00744-5)
Supplement: Supplementary file 1 — Supplementary information for All-optical modulation of quantum states by nonlinear metasurface [file 41377_2022_744_MOESM1_ESM.pdf]

Supplementary information for "All-optical modulation of quantum states by nonlinear metasurface"

Di Zhang,[1] Yang Chen,[2,3] Shengchao Gong,[1] Wei Wu,[1] Wei Cai,[1] Mengxin Ren,[1,4,a)] Xifeng Ren,[2,3,b)] Shuang Zhang,[5] Guangcan Guo,[2,3] and Jingjun Xu[1,c)]

[1)] *The Key Laboratory of Weak-Light Nonlinear Photonics, Ministry of Education, School of Physics and TEDA Applied Physics Institute, Nankai University, Tianjin, 300071, China*

[2)] *Key Laboratory of Quantum Information, CAS, University of Science and Technology of China, Hefei, 230026, China*

[3)] *Synergetic Innovation Center of Quantum Information & Quantum Physics, University of Science and Technology of China, Hefei, 230026, China*

[4)] *Collaborative Innovation Center of Extreme Optics, Shanxi University, Taiyuan, Shanxi 030006, China*

[5)] *Department of Physics, The University of Hong Kong, Hong Kong, China*

[a)] Electronic mail: ren_mengxin@nankai.edu.cn

[b)] Electronic mail: renxf@ustc.edu.cn

[c)] Electronic mail: jjxu@nankai.edu.cn

I. SPECTRAL MEASUREMENTS OF SAMPLE

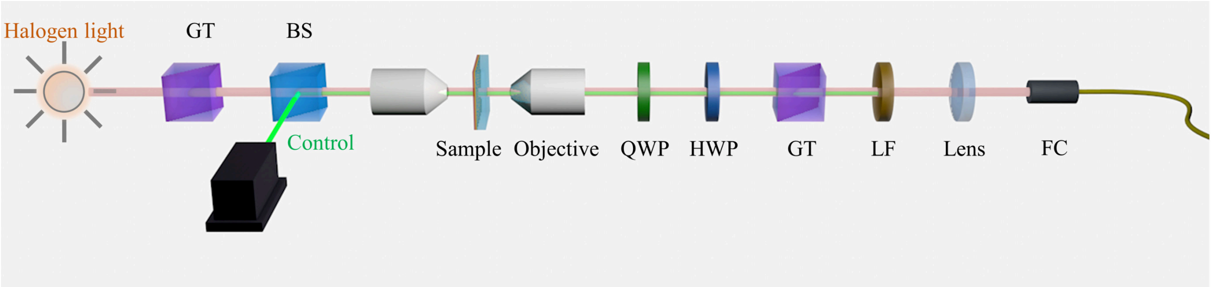

FIG. S1. **Experimental setup to measure classical optical spectra.** Transmission spectra T of metasurface for V- and H-polarized incidence were measured with halogen light illumination and were recorded by a multi-mode fiber coupled spectrometer. To determine the relative phase ϕ between the transmitted V- and H-polarizations, the incident light was further set along $+45°$ direction. And using a combination of QWP, HWP and GT, the transmitted light was decomposed into the $\pm45°$, left- and right-handed circular polarization components, whose spectra were measured successively. To introduce nonlinear optical stimulation, a 532 nm laser was combined with the halogen light by a polarization-independent BS. GT: Glan-Taylor polarizer; BS: beam splitter; QWP: quarter-waveplate; HWP: half-waveplate; LF: long-pass filter; FC: fiber coupler.

We measured transmission spectra of sample under V- and H-linear polarizations using a home-made spectrometer system, as shown in Fig. S1. A halogen lamp was used as light source, and two objectives (20×, 0.40 N.A. and 20×, 0.45 N.A.) were used to illuminate the sample and collect the transmitted signal, respectively. The transmitted light was analyzed by a multi-mode fiber coupled spectrometer (HR4000, Ocean Optics). The relative phase ϕ between the transmitted V- and H-polarizations was measured by further setting the incidence along $+45°$ direction. And the transmitted light was decomposed into $\pm45°$ (labeled as $T_{\pm45}$), left- and right-handed circular (T_L and T_R) polarization components successively by different orientation combinations of QWP, HWP and GT on the transmitted side (as detailed in Table S1), and the corresponding spectra were measured separately. Thus, ϕ can be calculated via $\phi = \arctan \frac{T_L - T_R}{T_{+45} - T_{-45}}$. A 532 nm laser was aligned with the halogen light by a polarization-independent beam splitter (BS), which stimulated the sample to nonlinearly control the transmission spectra. The results of the nonlinear controlled spectra for T and ϕ are shown in Fig. 2 in the main text. The linear absorption spectra are shown in Fig. S2.

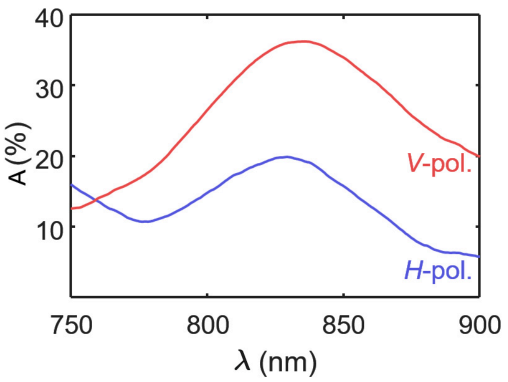

FIG. S2. **Absorption spectra of the metasurface.** The blue line corresponds to H-polarized light and the red line is V-polarized light.

TABLE S1. Decomposition of transmitted light by different combinations of QWP, HWP and GT

QWP	HWP	GT (analyzer)	Analyzed basis
45°	0°	0°	left-circular polarization (T_L)
45°	45°	0°	right-circular polarization (T_R)
45°	22.5°	0°	+45° polarization (T_{+45})
45°	−22.5°	0°	−45° polarization (T_{-45})

II. SIMULATION OF THE SPECTRA OF METASURFACE

The refractive indices of Au, SiO_2, and ethyl red used in the simulation are shown in Fig. S3.

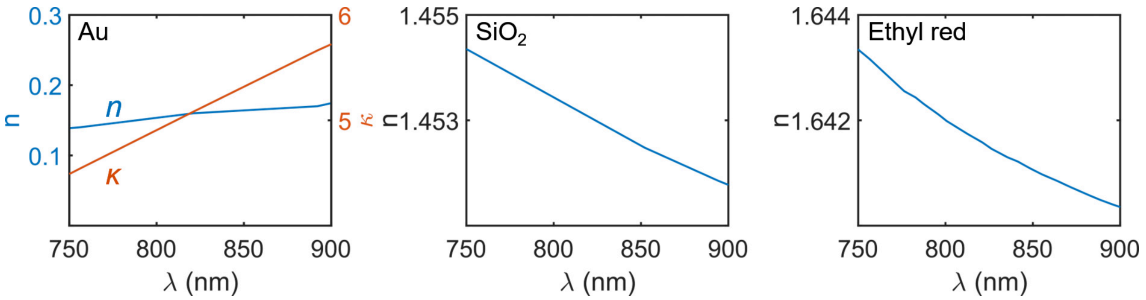

FIG. S3. **The refractive indices of Au, SiO_2, and ethyl red film used in simulations.**

The spectra of the metasurface between the simulation and experiment show similar

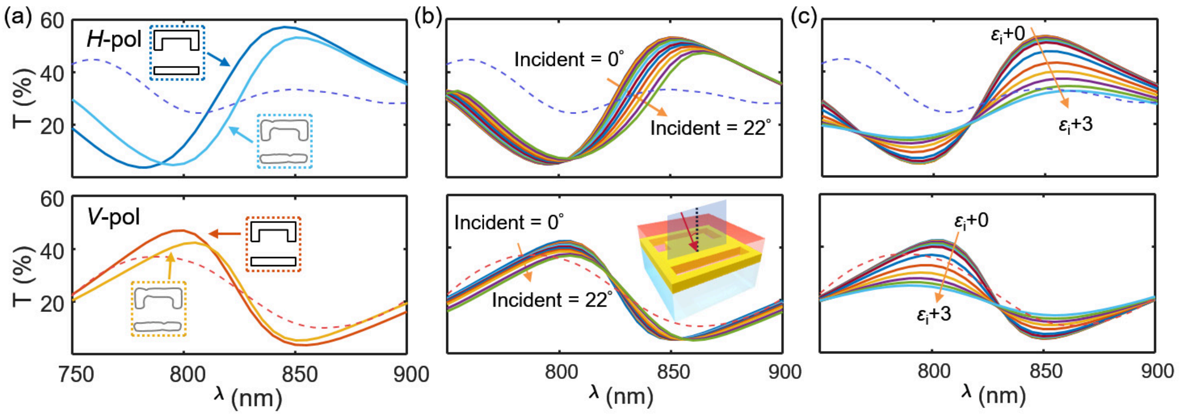

FIG. S4. The simulated transmission spectra when considering **(a)** the difference between ideal rectangular slits (dark-blue and dark-red curves) and the roundness of the structures (light-blue and light-yellow curves), **(b)** the different incident angles of the incident light, and **(c)** the increase of the imaginary part of the dielectric permittivity of Au. Dashed lines are experimental spectra replotted from the main text.

resonant characters (the positions of peaks and valleys), however, the amplitudes of the experimental results are flattened than simulations. We conducted several simulations, and the following aspects may explain such flattened spectra: (1) The geometric profile. The dark-blue and dark-red lines in Fig. S4(a) present the simulated spectra for the slits with ideal rectangular profiles. The light-blue and light-yellow curves give the results that take into account the actual shape of the slits according to the SEM images. The latter ones show slight red-shift and decreased resonance magnitude compared with the results of the ideal profiles. However, discrepancy still exists between the simulated results based on actual geometry and the experimental spectra (replotted here by dashed curves). (2) The incident angles. The halogen lamp light was focused on the metasurface by an objective (20×, 0.4 N.A.) in the measurement of the spectra, thus the metasurface was not illuminated by normally incident plane wave, but rather by a convergent light cone with multiple incident angles. We calculated the transmission spectra under various incident angles, as shown in Fig. S4(b). As the incident angle increases, the spectra are red-shifted and the resonance magnitudes decrease. However, there is still a discrepancy from the experimental spectra (dashed curves). (3) The dielectric permittivity of Au. The metallic metasurface was fabricated by FIB milling using a Gallium (Ga^+) ion beam. The Ga^+ ions inevitably

implant and accumulate in the metal layer, causing undesired contamination and absorption. Furthermore, surface scattering and grain boundary effects in the gold layer may also increase the losses of the metal layer.[1] These would induce the increase in the imaginary part of the permittivity of Au.[2] As shown in Fig. S4(c), as the imaginary part of the permittivity of Au ε_i increases, the transmittance spectra apparently flattened, and tend to match the experimental results.

III. CHARACTERIZATION OF ENTANGLED PHOTON PAIRS

The experimental setup is shown in Fig. 3(a) of the main text. The measured coincident photon counts in each polarization base are shown in Table S2. We performed quantum state tomography (QST) to characterize the entanglement properties of photon pairs. We used standard QST followed by maximum likelihood estimation (MLE) to reconstruct the density matrix ρ of the state, which was proposed by D. F. V. James, et al. in Ref. [3]. The density matrix of two-photon state can be expressed through 2-photon Stokes parameters $S_{i1,i2}$ and Pauli operators σ_i:

$$\rho_{\text{QST}} = \frac{1}{4} \sum_{i1,i2=0}^{3} \frac{S_{i1,i2}}{S_{0,0}} \sigma_{i1} \otimes \sigma_{i2}, \tag{S1}$$

where the single-qubit measurement operators μ_i relate with Pauli matrices by

$$\mu_i = \sum_{j=0}^{3} Y_{i,j} \sigma_j, \tag{S2}$$

where $Y_{i,j}$ are i, j-th elements of

$$Y = \begin{pmatrix} 1 & 0 & 0 & 0 \\ \frac{1}{2} & \frac{1}{2} & 0 & 0 \\ \frac{1}{2} & 0 & \frac{1}{2} & 0 \\ \frac{1}{2} & 0 & 0 & \frac{1}{2} \end{pmatrix}. \tag{S3}$$

And the photon counts $n_{i1,i2}$ in various polarization-projection measurements are related with $S_{i1,i2}$ by

$$S_{i1,i2} = \sum_{j1,j2=0}^{3} Y^{-1}_{i1,j1} Y^{-1}_{i2,j2} n_{i1,i2}, \tag{S4}$$

TABLE S2. Coincident photon counts in each measurement polarization base with the relationship with the control light power P_C

**Base**	**0 mW**	**2 mW**	**4 mW**	**6 mW**	**8 mW**	**10 mW**	**12 mW**	**14 mW**	**16 mW**	**18 mW**	**20 mW**
$H_S H_I$	377	389	428	430	447	530	634	787	772	756	692
$V_S H_I$	3	3	3	3	2	4	1	3	1	3	4
$D_S H_I$	179	185	189	194	228	259	302	340	363	345	341
$R_S H_I$	167	171	188	193	229	253	315	345	372	395	356
$H_S V_I$	1	3	5	6	8	9	6	6	5	5	10
$V_S V_I$	775	861	810	809	787	774	739	743	681	631	547
$D_S V_I$	370	358	365	354	330	366	343	327	311	242	240
$R_S V_I$	451	459	444	414	432	446	387	440	377	324	302
$H_S D_I$	183	160	183	211	225	232	249	331	334	351	310
$V_S D_I$	406	395	377	375	394	385	339	364	302	255	252
$D_S D_I$	491	524	546	508	590	574	630	663	629	576	581
$R_S D_I$	411	382	429	372	402	417	415	418	386	343	322
$H_S R_I$	143	150	161	193	190	237	313	307	338	330	336
$V_S R_I$	412	463	397	400	432	432	409	382	353	289	270
$D_S R_I$	389	356	358	352	345	332	367	372	369	331	324
$R_S R_I$	62	51	42	37	37	23	16	10	10	8	9

where $Y^{-1}_{i,j}$ are elements of

$$Y^{-1} = \begin{pmatrix} 1 & 0 & 0 & 0 \\ -1 & 2 & 0 & 0 \\ -1 & 0 & 2 & 0 \\ -1 & 0 & 0 & 2 \end{pmatrix}. \tag{S5}$$

By substituting the selected measurement bases and photon counts in the above formulas, the density matrix ρ_{QST} can be reconstructed. This will always work when assuming perfect

experimental conditions. However, in reality, it may produce results that violate basic properties, such as positivity. The MLE is then used to avoid this problem.

The MLE generally contains the following steps.

(1) Generating a 'physical' density matrix $\rho_p = (T^\dagger T)/\mathrm{Tr(T^\dagger T)}$ that matches the condition of normalization, Hermiticity, and positivity. Where T shows:

$$T = \begin{pmatrix} t_1 & 0 & 0 & 0 \\ t_5 + it_6 & t_2 & 0 & 0 \\ t_{11} + it_{12} & t_7 + it_8 & t_3 & 0 \\ t_{15} + it_{16} & t_{13} + it_{14} & t_9 + it_{10} & t_4 \end{pmatrix} = \begin{pmatrix} \sqrt{\frac{\Delta}{M_{11}^{(1)}}} & 0 & 0 & 0 \\ \frac{M_{12}^{(1)}}{\sqrt{M_{11}^{(1)} M_{11,22}^{(2)}}} & \sqrt{\frac{M_{11}^{(1)}}{M_{11,22}^{(2)}}} & 0 & 0 \\ \frac{M_{12,23}^{(2)}}{\sqrt{\rho_{44} M_{11,22}^{(2)}}} & \frac{M_{11,23}^{(2)}}{\sqrt{\rho_{44} M_{11,22}^{(2)}}} & \sqrt{\frac{M_{11,22}^{(2)}}{\rho_{44}}} & 0 \\ \frac{\rho_{41}}{\sqrt{\rho_{44}}} & \frac{\rho_{42}}{\sqrt{\rho_{44}}} & \frac{\rho_{43}}{\sqrt{\rho_{44}}} & \sqrt{\rho_{44}} \end{pmatrix}, \quad \text{(S6)}$$

in which ρ_{ij} is the element in the density matrix ρ_{QST} reconstructed in the QST process; $M_{ij}^{(1)}$ is the determinant of the 3×3 matrix formed by deleting the ith row and jth column of ρ_{QST}; $M_{ij,kl}^{(2)}$ is the determinant of the 2×2 matrix formed by deleting the ith and kth rows and jth and lth columns of ρ_{QST}; and $\Delta = \mathrm{Det}(\rho_{\mathrm{QST}})$.

(2) Introducing a 'likelihood function':

$$L = \sum_{\nu=1}^{16} \frac{\left[N \langle \psi_\nu | \rho_p | \psi_\nu \rangle - n_\nu\right]^2}{2N \langle \psi_\nu | \rho_p | \psi_\nu \rangle}, \quad \text{(S7)}$$

in which $|\psi_\nu\rangle$ are states of the measurement bases. The task is then an optimization problem to find the minimum of the L function, which represents the 'likelihood' that the matrix ρ_p could produce the measured data n_ν.

(3) Numerical optimization. We set ρ_{QST} to determine the initial set of values for $t_1, t_2, ..., t_{16}$, and used standard numerical optimization technique to optimize the function L to find a minimum value.

Finally, we can find the optimized density matrix ρ_p as the measured density matrix ρ.

The concurrence is defined by $C = \max\{\sqrt{\nu_1} - \sqrt{\nu_2} - \sqrt{\nu_3} - \sqrt{\nu_4}, 0\}$, where ν_i ($i = 1, 2, 3, 4$, $\nu_1 \geq \nu_2 \geq \nu_3 \geq \nu_4$) are eigen values of the matrix $M = \rho \times \sigma \times \rho^T \times \sigma$ (the superscript T represents the transpose of the matrix, $\sigma = \sigma_y \otimes \sigma_y$ is the 'spin flip matrix', and $\sigma_y = \begin{pmatrix} 0 & -i \\ i & 0 \end{pmatrix}$ is one of the Paul matrices). The fidelity of the experimental result relative to the Bell state is defined as $F = \mathrm{Tr}\left\{\rho \left|\Psi_0\right\rangle \left\langle\Psi_0\right|\right\}$, where Ψ_0 is the Bell state, and $\mathrm{Tr}\{\}$ represents the trace of the matrix.

IV. TUNING BEHAVIORS OF NONLINEAR METASURFACE

The numerically simulated potential tuning behaviors are shown in Fig. S5. The black curves exemplify how the quantum state evolves when the refractive index change of the nonlinear polymer film varies from $\Delta n_{\mathrm{ER}} = 0$ to -0.5. The concurrence and fidelity of the initial quantum state (point of $\Delta n_{\mathrm{ER}} = 0$) are much lower than 100%, and then they gradually increase as the refractive index decreases. When $\Delta n_{\mathrm{ER}} = -0.05$, the quantum state recovers, and the concurrence and fidelity are about 100%, which corresponds to the expected distilled state. As the refractive index Δn_{ER} further decreases, the entangled state is over-tuned, and the concurrence and fidelity drop.

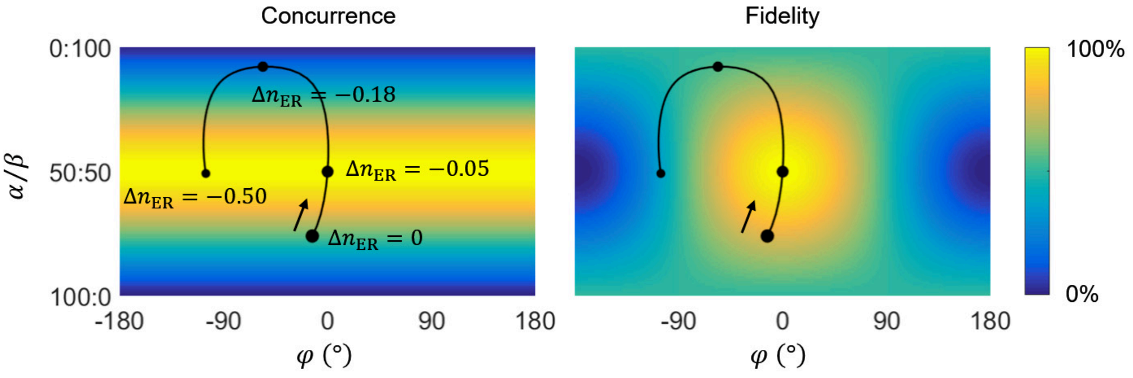

FIG. S5. **Simulated tuning curve of the entangled state.** The refractive index change of the ethyl red film varies between $\Delta n_{\mathrm{ER}} = 0$ to -0.5. The concurrence and the fidelity of the entangled state first increase and then drop as the refractive index n_{ER} decreases. The quantum state is distilled when $\Delta n_{\mathrm{ER}} = -0.05$. φ is the phase difference between $|V_S\rangle\,|V_I\rangle$ and $|H_S\rangle\,|H_I\rangle$.

The quantum states located on the black curve in Fig. S5, with the variation of the refractive index of ethyl red Δn_{ER} between 0 and -0.05, can be distilled by our metasurface design used in the main text. Furthermore, our nonlinear metasurface platform can be applied to distilling other quantum states. For example, by scaling the geometric dimensions of the metaatoms (both the slits' length and width) by a factor m (varies from 0.90 to 1.08 with the step of 0.02), different quantum states can be distilled by $\Delta n_{\mathrm{ER}} = -0.05$, as shown in Fig. S6.

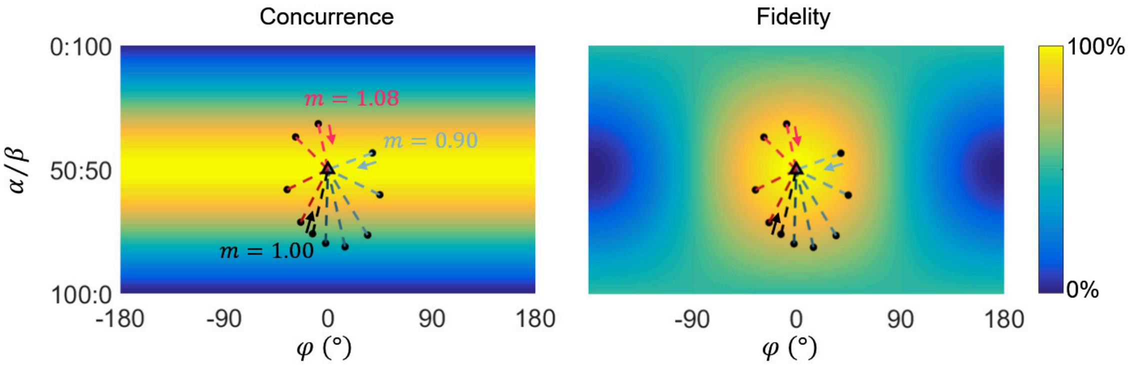

FIG. S6. **Simulated results of distilling various non-maximally entangled states using nonlinear metasurface.** The geometric parameters of the metasurface are scaled by a factor m. Black dots represent the initial states, and the black triangle in the center is the distilled state with the refractive index change of the nonlinear polymer film $\Delta n_{\mathrm{ER}} = -0.05$. The dashed lines represent the 'distillation path' from the initial states towards the final maximally entangled state.

V. TEMPORAL RESPONSE OF NONLINEAR METASURFACE

To characterize the temporal response of the metasurface, the control light was mechanically chopped, thereby inducing periodic modulation over the signal light. The QWP, HWP, and GT served as the polarization analyzer, and were properly oriented to eliminate the transmitted signal light at the initial state. When the control light illuminated the metasurface, the polarization of the transmitted signal light was changed due to the different variations in transmittance between the horizontal and the vertical polarization components, which subsequently induced modulation in the leaked light intensity, which was monitored by an oscilloscope. Figure S7 shows the result of the dynamic response of our metasurface. Using the biexponential fitting,[4] the rise and decay rates of the fast (and slow) components are obtained as 0.61 (and 13.80) and 0.24 (and 27.83) ms, respectively.

VI. REPRODUCIBILITY OF NONLINEAR SPECTRAL MEASUREMENTS

We fabricated another metasurface array and reperformed the nonlinear spectral measurements, as shown in Fig. S8. The results are reasonably agreed with Fig. 2(b) in the main text, confirming the well reproducibility of the experiments.

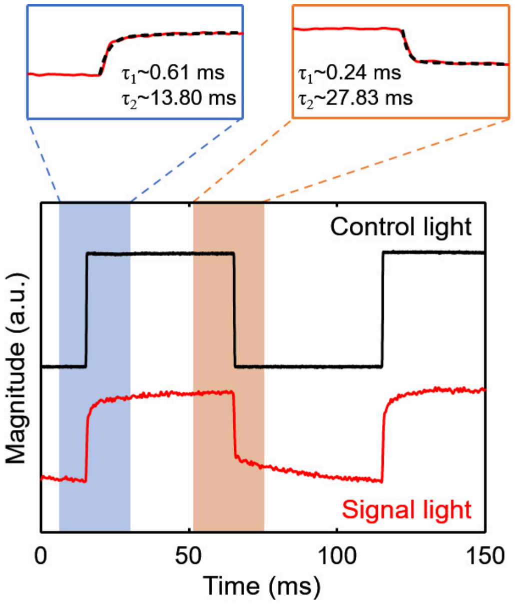

FIG. S7. **Dynamic response of the nonlinear metasurface.** The solid black line shows the temporal profile of the chopped control light, and the red lines show the dynamic response of the signal light. Dashed black lines in the top two figures are biexponential fitted eye-guides.

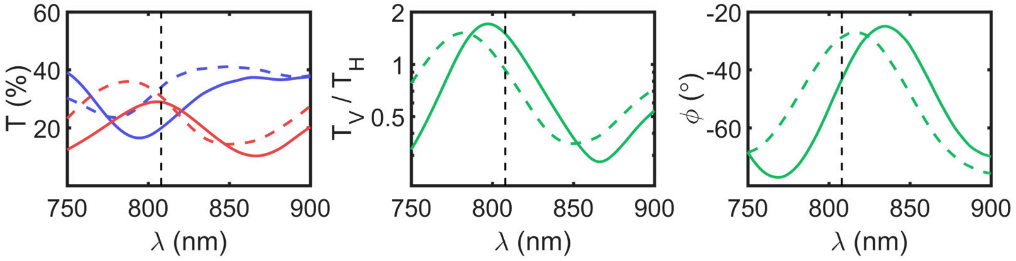

FIG. S8. **Nonlinear spectra of a newly fabricated metasurface.**

REFERENCES

[1] S. S. Kruk, Z. J. Wong, E. Pshenay-Severin, K. O'brien, D. N. Neshev, Y. S. Kivshar, and X. Zhang, "Magnetic hyperbolic optical metamaterials," Nat. Commun. **7**, 11329 (2016).

[2] L. Ouyang, D. Rosenmann, D. A. Czaplewski, J. Gao, and X. Yang, "Broadband infrared circular dichroism in chiral metasurface absorbers," Nanotechnology **31**, 295203 (2020).

[3] D. F. V. James, P. G. Kwiat, W. J. Munro, and A. G. White, "Measurement of qubits," Phys. Rev. A **64**, 052312 (2001).

[4]M.-X. Ren, W. Wu, W. Cai, B. Pi, X.-Z. Zhang, and J.-J. Xu, "Reconfigurable metasurfaces that enable light polarization control by light," Light Sci. Appl. **6**, e16254 (2017).
